# One-Step, Three-Factor Passthought Authentication With Custom-Fit, In-Ear EEG

**DOI:** 10.3389/fnins.2019.00354

**Published:** 2019-04-30

**Authors:** Nick Merrill, Max T. Curran, Swapan Gandhi, John Chuang

**Affiliations:** ^1^BioSENSE, School of Information, University of California, Berkeley, Berkeley, CA, United States; ^2^Starkey Hearing Research Center, Berkeley, CA, United States

**Keywords:** passthoughts, authentication, in-ear EEG, ubiquitous EEG, brain-computer interface, usable security, human-computer interaction

## Abstract

In-ear EEG offers a promising path toward usable, discreet brain-computer interfaces (BCIs) for both healthy individuals and persons with disabilities. To test the promise of this modality, we produced a brain-based authentication system using custom-fit EEG earpieces. In a sample of *N* = 7 participants, we demonstrated that our system has high accuracy, higher than prior work using non-custom earpieces. We demonstrated that both inherence and knowledge factors contribute to authentication accuracy, and performed a simulated attack to show our system's robustness against impersonation. From an authentication standpoint, our system provides three factors of authentication in a single step. From a usability standpoint, our system does not require a cumbersome, head-worn device.

## 1. Introduction

The hardware that drives EEG-based BCIs has improved dramatically over the past 5 years, decreasing in size and cost by orders of magnitude (Grierson and Kiefer, 2011). Many consumer devices leverage this technology: as of December 2018, there are at least seven EEG devices on the market, ranging from 100 to 500 USD, and featuring one to sixteen electrodes. Many of them transmit data wirelessly to computers and smart devices. Meanwhile, advances in machine learning have radically improved the reliability of BCI applications. Taken together, prospects seem bright for the wider adoption of BCIs in everyday life.

However, the head-worn form-factor, and awkward visibility of EEG-based BCIs has proven a stubborn challenge to BCI adoption (Mihajlovic et al., 2015). Both disabled and healthy subjects complain about the comfort of head-worn devices, the difficulty of applying electrodes correctly to the scalp, and questionable aesthetics of wearing such a visible device in public, social settings (Ekandem et al., 2012; David Hairston et al., 2014).

One possible solution to this problem is to embed EEG electrodes in earbuds, collecting EEG signals from the ear canal. While early work framed in-ear EEG largely as a tradeoff between ergonomics and signal quality (Kidmose et al., 2013), in-ear EEG signals are at least robust enough to detect auditory evoked responses (Kidmose et al., 2012), and more recent work has indicated that EEG collected in the ear may have its own, unique affordances. For example, one study built a rudimentary eye-tracker using ocular signals (EOG, or electrooculography) collected from the ear canal (Manabe and Fukumoto, 2013).

**Figure 1:**
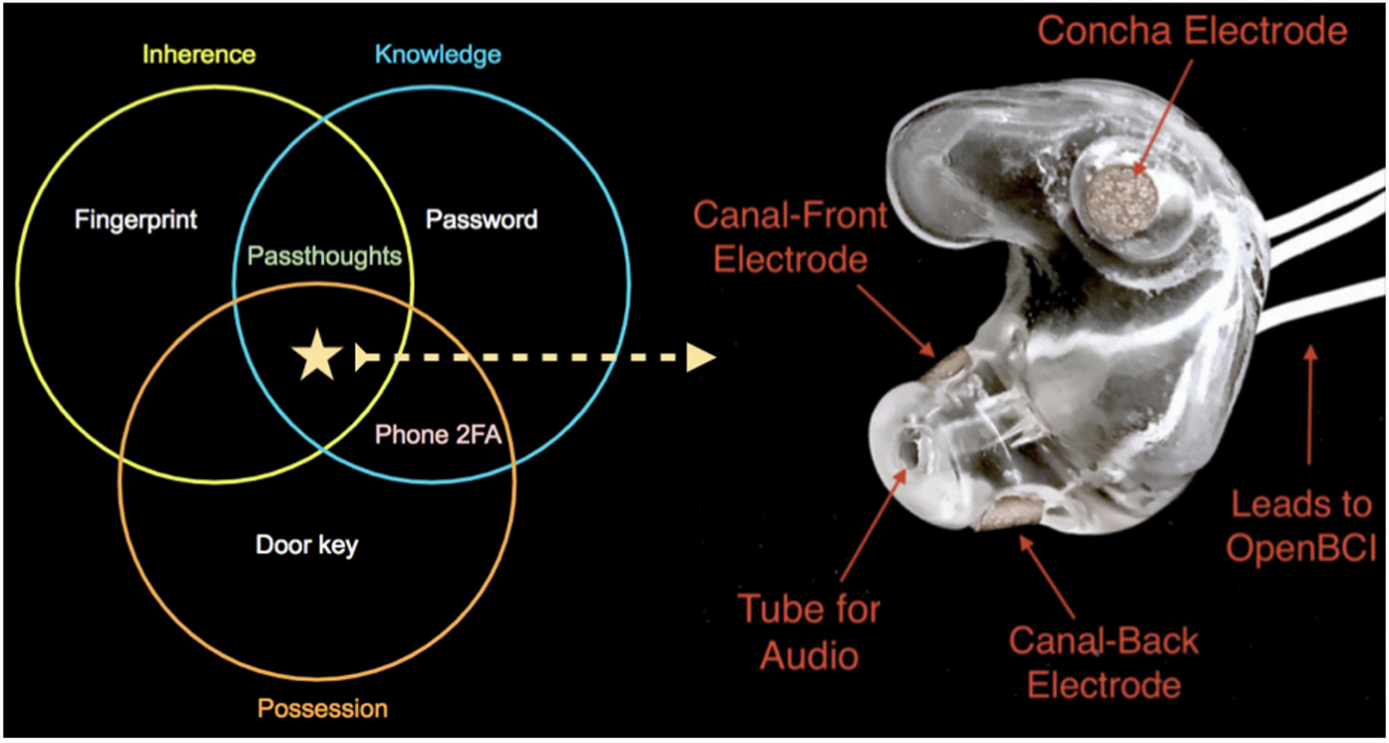
On the right, one of the manufactured custom-fit earpieces with three embedded electrodes located in the concha, front-facing (anterior) in the ear canal, and back-facing (posterior) in the ear canal. On the left, three factors of authentication. Passthoughts authentication with a custom-fit in-ear EEG satisfies all three factors.

To test in-ear EEG's capacity to produce usable BCI applications, this paper attempts to use the sensing modality to construct a brain-based authentication system (Chuang et al., 2013) using custom-fit, EEG earbuds. Authentication relies on one or more *factors*: knowledge (something one knows), posesssion (something one has), or inherence (properties of one's body). Where multifactor authentication provides added security over single-factor authentication such as passwords, multiple factors typically require multiple steps (e.g., entering a password, then entering a code from one's cellphone). One particular brain-based authentication strategy, *passthoughts*, combines multiple factors of authentication into a single step: a knowledge factor (one's secret thought), and a biometric factor (the unique way one express that thought neurally) (Chuang, 2014). By incorporating a custom-fit earbud, we set out to combine all three factors of authentication into a single step (Figure 1).

This paper makes several, distinct contributions. First, we achieve a 99.82% authentication accuracy with zero false acceptance rate (FAR) using personalized custom-fit three-channel EEG earpieces and a passthoughts authentication paradigm. Second, we quantify the improvements over prior art in authentication accuracy due to the use of custom-fit vs. generic earpieces, and the use of multiple electrodes vs. a single electrode. Third, we evaluate multiple classification strategies that allows us to compare the relative contributions of the inherence factor and knowledge factor to authentication accuracy. Fourth, we perform simulation attacks to demonstrate the method's robustness against impersonation via four scenarios where the attacker has access to the target's earpiece and/or secret passthoughts.

Collectively, we build a case that in-ear EEG could offer a viable, usable road to accurate BCI applications, for healthy individuals or persons with disabilities. In addition, we argue that passthoughts authentication using personalized custom-fit earpieces offers a viable and attractive path toward one-step three-factor authentication.

## 2. Related Work

### 2.1. In-Ear EEG

The concept of in-ear EEG was introduced in 2011 with a demonstration of the feasibility of recording brainwave signals from within the ear canal (Looney et al., 2011). The in-ear placement can produce signal-to-noise ratios comparable to those from conventional EEG electrode placements, is robust to common sources of artifacts, and can be used in a brain-computer interface (BCI) system based on auditory and visual evoked potentials (Kidmose et al., 2013). One previous study attempted to demonstrate user authentication using in-ear EEG, but was only able to attain an accuracy level of 80%, limited by the use of a consumer-grade device with a single generic-fit electrode (Curran et al., 2016). A follow-up study with a single, generic-fit electrode achieved an accuracy of 95.7% over multiple days (Nakamura et al., 2018).

### 2.2. Passthoughts and Behavioral Authentication

The use of EEG as a biometric signal for user authentication has a relatively short history. In 2005, Thorpe et al. motivated and outlined the design of a passthoughts system (Thorpe et al., 2005). Since 2002, a number of independent groups have achieved 99–100% authentication accuracy for small populations using research-grade and consumer-grade scalp-based EEG systems (Poulos et al., 2002; Marcel and Millan, 2007; Ashby et al., 2011; Chuang et al., 2013). Several recent works on brainwave biometrics have independently demonstrated individuals' EEG permanence over 1–6 months (Armstrong et al., 2015; Maiorana et al., 2016) or even over 1 year (Ruiz-Blondet et al., 2017).

#### 2.2.1. Authentication Factors

Behavioral authentication methods such as keystroke dynamics and speaker authentication can be categorized as one-step two-factor authentication schemes. In both cases, the knowledge factor (password or passphrase) and inherence factor (typing rhythm or speaker's voice) are employed (Monrose and Rubin, 1997). In contrast, the Nymi band supports one-step two-factor authentication via the inherence factor (cardiac rhythm that is supposed to be unique to each individual) and the possession factor (the wearing of the band on the wrist) (Nymi, 2017). However, as far as we know, no one has proposed or demonstrated a one-step three-factor authentication scheme.

### 2.3. Usable Authentication

When proposing or evaluating authentication paradigms, robustness against imposters is often a first consideration, but the usability of these systems is of equal importance as they must conform to a person's needs and lifestyle to warrant adoption and prolonged use. Sasse et al. describe usability issues with common knowledge-based systems like alphanumeric passwords, in particular that a breach in systems which require users to remember complex passwords that must be frequently changed is a failure on the part of the system's design, not the fault of the user (Sasse et al., 2001). Other research analyzed some of the complexities of applying human factors heuristics for interface design to authentication, and indicate the importance of social acceptability, learnability, and simplicity of authentication methods (Braz and Robert, 2006). Technologies worn on the head entail particular usability issues; in their analysis of user perceptions of headworn devices, Genaro et al. identified design, usability, ease of use, and obtrusiveness among the top ten concerns of users, as well as qualitative comments around comfort and “looking weird” (Genaro Motti and Caine, 2014).

Mobile and wearable technologies' continuous proximity to the user's body provides favorable conditions for unobtrusively capturing biometrics for authentication. Many such uses have been proposed that embrace usability like touch-based interactions (Holz and Knaust, 2015; Tartz and Gooding, 2015) and walking patterns (Lu et al., 2014) using mobile phones, as well as identification via head movements and blinking in head-worn devices (Rogers et al., 2015). However, these typically draw only from the inherence factor. Chen et al. proposed an inherence and knowledge two-factor method for multi-touch mobile devices based on a user's unique finger tapping of a song (Chen et al., 2015), though it may be vulnerable to “shoulder surfing”: imposters observing and mimicking the behavior to gain access.

### 2.4. One-Step, Three-Factor Authentication

It is well appreciated by experts and end-users alike that strong authentication is critical to cybersecurity and privacy, now and into the future. Unfortunately, news reports of celebrity account hackings serve as regular reminders that the currently dominant method of authentication in consumer applications, single-factor authentication using passwords or other user-chosen secrets, faces many challenges. Many major online services have strongly encouraged their users to adopt two-factor authentication (2FA). However, submitting two different authenticators in two separate steps has frustrated wide adoption due to its additional hassle to users. Modern smartphones, for instance, already support device unlock using either a user-selected passcode or a fingerprint. These devices could very well support a two-step two-factor authentication scheme if desired. However, it is easy to understand why users would balk at having to enter a passcode *and* provide a fingerprint each time they want to unlock their phone.

“One-step two-factor authentication” has been proposed as a new approach to authentication that can provide the security benefits of two-factor authentication without incurring the hassle cost of two-step verification (Chuang, 2014). In this work we undertake, to the best of our knowledge, the first-ever study and design of one-step, *three*-factor authentication. In computer security, authenticators are classified into three types: knowledge factors (e.g., passwords and PINs), possession factors (e.g., physical tokens, ATM cards), and inherence factors (e.g., fingerprints and other biometrics). By taking advantage of a physical token in the form of personalized earpieces, the uniqueness of an individual's brainwaves, and a choice of mental task to use as one's “passthought,” we seek to achieve all three factors of authentication within a single step by the user.

In the system we propose here we seek to incorporate recommendations from this research for improved usability while maintaining a highly secure system. The mental tasks we test are simple and personally relevant; instead of complex alphanumeric patterns like a traditional password, a mental activity like relaxed breathing or imagining a portion of one's favorite song are easy for a user to remember and perform as shown by participant feedback in previous passthoughts research and in our own results later in this paper. These mental activities are largely invisible to “shoulder surfing” attempts by onlookers, and furthermore present a possible solution to “rubber-hose attacks” (forceful coercion to divulge a password); a thought has a particular expression unique to an individual, the specific performance of which cannot be described and thus cannnot be coerced or forcibly unlike for example the combination to a padlock or fingerprint. Finally, to combat the wearability and obtrusiveness issues of scalp-based EEG systems used in other brain-based authentication research, our system's form factor of earpieces with embedded electrodes is highly similar to earbud headphones or wireless headsets already commonly worn and generally socially accepted technologies.

## 3. Methods

### 3.1. Study Overview

Seven male, right-handed participants (P1–P7), five students and two researchers, were recruited via a university mailing list and completed our study protocol approved by our local ethics review board. The two researcher participants were also involved in the development of this study. Though this sample is relatively homogenous and greater diversity is necessary for a larger real-world feasibility assessment, this quality interestingly functions to strengthen the results of a system designed to discriminate between users (see Discussion). After participants' 3D ear molds were obtained, the custom-fit earpieces were manufactured, and their fit and electrical impedances were checked, we proceeded to the collection of study data.

Data collection consisted of participants completing a demographics questionnaire, a setup period with the OpenBCI system and earpieces sed for EEG collection with a second impedance check, their performance of nine mental tasks, and finally a post-experiment questionnaire.

### 3.2. Earpiece Design and Manufacturing

Earpieces were produced by an audiologist at Starkey, a manufacturer of hearing aids. To produce custom ear impressions, subjects' ears were cleaned, a cotton ball with a string attached was placed inside the ear canal, and silicone was injected into the canals. Starkey “Precise3S Classic” two-part silicone impression material was used. When the silicone dried after a few minutes, the string was pulled to remove the impression from the ear canal. This impression was then scanned with a 3D scanner, and the resulting scan modified to achieve a comfortable fit and to ensure the intended electrode sites would make good contact with the skin. Channels were created in the 3D model to allow wire leads and associated EEG electrodes as well as a plastic tube to deliver audio. This 3D model was then sent to a 3D printer after which wires, leads, and associated AgCl electrodes were installed. Cortech EC-DC-AGP1 electrodes were used for the canal electrodes, and Cortech EC-DC-AGE6 electrodes were used for the concha electrode. The positions of the earpiece electrodes were simplified from those described in Mikkelsen et al. (2015). We reduced the number of canal electrodes in order to prevent electrical bridging and positioned them approximately 180° apart in the canal (posterior/back and anterior/front locations in the canal). One other electrode was placed in the concha. An example of one of the manufactured earpieces is shown in Figure 1.

The electrodes were purchased from Cortech:

Canal electrodes: https://cortechsolutions.com/product/ec-dc-agp1/

Concha electrode: https://cortechsolutions.com/product/ec-dc-age6/

### 3.3. Mental Tasks

We selected a set of mental tasks based on findings in related work regarding the relative strengths of different tasks in authentication accuracy and usability as reported by participants (Chuang et al., 2013; Curran et al., 2016). Furthermore, given the in-ear placement of the electrodes and therefore the proximity to the temporal lobes containing the auditory cortex, we tested several novel authentication tasks based specifically on aural imagery or stimuli. The nine authentication tasks and their attributes are listed in Table 1. Our strategy was to select tasks that captured a diversity across dimensions of external stimuli, involving a personal secret, eyes open or closed (due to known effects on EEG), and different types of mental imagery.

**Table 1:** The nine authentication tasks and their properties.

**Task**	**Description**	**Stimuli?**	**Secret?**	**Imagery**
Breathe	Relaxed breathing	No	No	None
Breathe - Open	Relaxed breathing with eyes open	No	No	None
Sport	Imagine attempting a chosen physical activity	No	Yes	Motor
Song	Imagine hearing a song	No	Yes	Aural
Song - Open	Song task, with eyes open	No	Yes	Aural
Speech	Imagine a chosen spoken phrase	No	Yes	Aural
Listen	Listen to noise modulated at 40 Hz	Yes	No	None
Face	Imagine a chosen person's face	No	Yes	Visual
Sequence	Imagine a face, number, and word on cues with eyes open	Yes	Yes	Visual

*We selected tasks with a variety of different properties, but preferred tasks that did not require external stimuli, as the need to present such stimuli at authentication time could present challenges for usability and user security. Tasks were performed with the participant's eyes closed unless otherwise noted*.

### 3.4. Data Collection Protocol

All sites were cleaned with ethanol prior to electrode placement and a small amount of conductive gel was used on each electrode. For EEG recording we used an 8-channel OpenBCI system (Michalska, 2009) which is open-source and costs about 600 USD; an alternative to medical-grade EEG systems (which cost >20,000 USD), with demonstrated effectiveness (Frey, 2016). We chose OpenBCI for its flexibility: despite the broad availability of low-cost EEG sensors, no commercially-available sensor allowed us to build our own recording configuration with a custom number, and configuration, of electrodes.

The ground was placed at the center of the forehead, at AFz according to the 10–20 International Standard for Electrode Placement (ISEP), and reference on the left mastoid (behind the left ear). We chose the AFz ground location to minimize the chances that our measurement setup caused differences between readings from the left and right electrodes, , though future systems using one ear only should test relocating the ground to a site on one ear (e.g., the earlobe). Six channels were used for the three electrodes on each earpiece (shown in Figure 1). For the remaining two channels, one AgCl ring electrode was placed on the right mastoid for later re-referencing, and one at Fp1 (ISEP location above the left eye) to validate the data collected in the ears against a common scalp-based placement. Before beginning the experiment, the data from each channel was visually inspected using the OpenBCI interface by having the participant clench their jaw and blink. Audio stimuli were delivered through small tubes in the earpieces.

**Figure 2:**
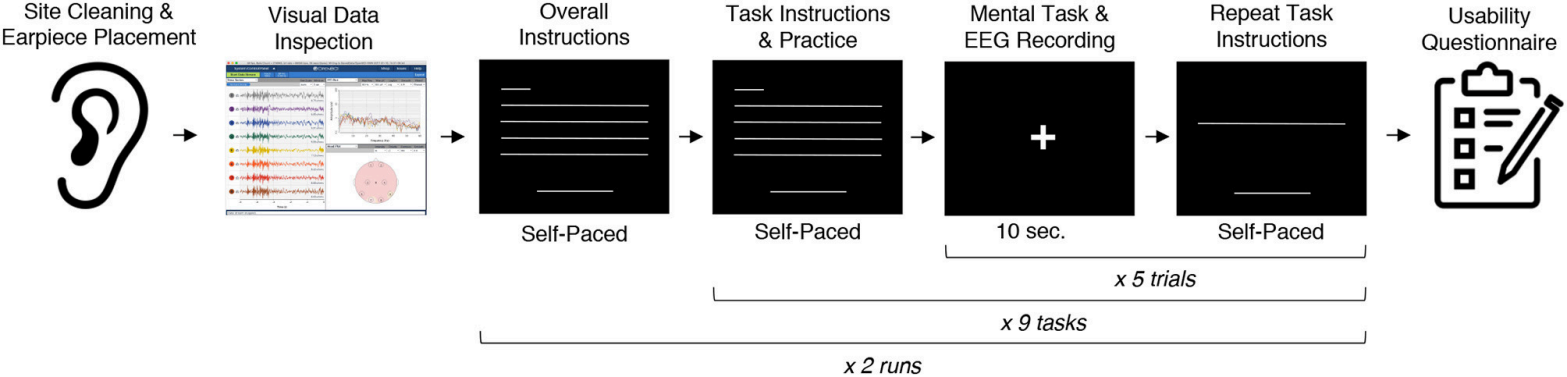
The data collection protocol. Approximately 2 weeks after data collection participants were contacted via e-mail to test recall of their passthoughts.

During the experiment, participants were seated in a comfortable position in a quiet room facing a laptop on which the instructions and stimuli were presented and timings recorded using PsychoPy (Peirce, 2007). All tasks were performed for five trials each, followed by another set of five trials each to reduce boredom and repetition effects. Each trial was 10 s in length, for a total of 10 trials or 100 s of data collected per task. This collection protocol is outlined in Figure 2. The instructions were read aloud to participants by the experimenter, and participants advanced using a pointer held in their lap to minimize motion artifacts in the data. The experimenter also recorded the participant's chosen secrets for the *sport, song, face, speech*, and *sequence* tasks and reminded the participant of these for the second set of trials. After EEG data collection, participants completed a usability questionnaire assessing each task on 7-point Likert-type scales on dimensions of ease of use, level of engagement, repeatability, and likeliness to use for real-world authentication as well as a few open response questions. Approximately 2 weeks after data collection participants were contacted via e-mail and asked to recall their choices for those tasks that involved chosen secrets.

## 4. Analysis

### 4.1. Data Validation

We confirm that the custom-fit earpieces were able to collect quality EEG data via two metrics: low impedances measured for the ear electrodes, and alpha-band EEG activity attenuation when a participant's eyes were open vs. closed.

It is important that the electrical impedances achieved for electrodes are low (<10 kOhm) to obtain quality EEG signals. Table 2 below summarizes the impedances across the seven participants' six ear channels. With the exception of a few channels in select participants, impedances achieved were good overall. Most of the recorded impedances of the earpiece electrodes were less than 5 kΩ, a benchmark used widely in previous ear EEG work, and all except two were less than 10 kΩ. Nonetheless, the data from all electrodes were tested in our other data quality test.

**Table 2:** Electrical impedances measured for concha (C), front (F), and back (B) earpiece electrodes.

	**Impedances [k**Ω**]**					
	**Left ear**			**Right ear**		
**P**	**C**	**F**	**B**	**C**	**F**	**B**
1	4	4	4	<1	4	3
2	9	5	4	3	4	4
3	4	5	4	9	6	9
4	4	5	4	3	16	9
5	9	20	7	3	7	9
6	5	8	2	1	1	9
7	2	9	8	7	5	6

**Figure 3:**
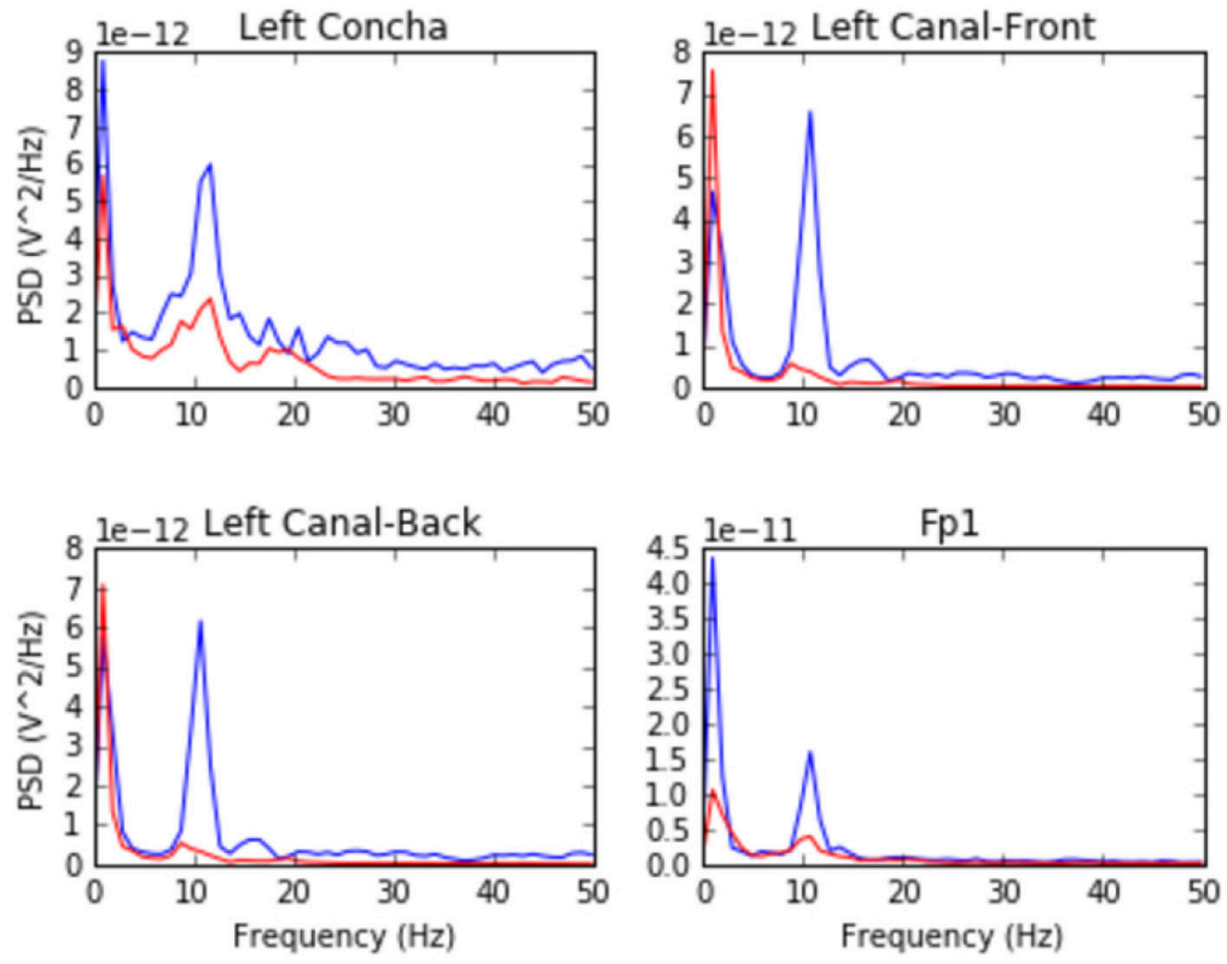
Alpha-attenuation (8–12 Hz range) in left ear and Fp1 channels, referenced at left mastoid. Red indicates breathing data with eyes open, blue indicates the same task with eyes closed.

For the alpha-attenuation test, data from the *breathe* task was compared with that of the *breathe - open* task. It is a well-known feature of EEG data that activity in the alpha-band (approximately 8–12 Hz) increases when the eyes are closed compared to when the eyes are open. This attenuation is clearly visible even in just a single trial's data from our earpieces and matches that seen in our Fp1 scalp electrode data. Figure 3 shows evidence of alpha attenuation in the left ear channels compared to Fp1, for one participant as an example. We see the same validation in the right ear channels.

### 4.2. Classification

Since past work has shown that classification tasks in EEG-based brain-computer interfaces (BCI) are linear (Garrett et al., 2003), we used XGBoost, a popular tool for logistic linear classification (Chen and Guestrin, 2016), to analyze the mental task EEG data. Compared to other linear classifiers, XGBoost uses gradient boosting in which an algorithm generates a decision tree of weak linear classifiers that minimizes a given loss function. Gradient boosting generally improves linear classification results without manually tuning hyper-parameters.

To produce feature vectors, we took slices of 100 raw values from each electrode (about 500 ms of data), and performed a Fourier transform to produce power spectra for each electrode during that slice. We concatenated all electrode power spectra together. No dimensionality reduction was applied. For each task, for each participant, 100 s of data were collected in total across 10 trials of 10 s each, resulting in 200 samples per participant, per task.

We trained the classifier such that positive examples were from the target participant and target task, and negative examples were selected randomly from any task from any other participant. From this corpus of positive and negative samples, we withheld one third of data for testing. The remaining training set was used to cross-validate an algorithm over 100 rounds on different splits of the data. The results of each cross-validation (CV) step was used to iteratively tweak classifier parameters.

For the predictions, the evaluation regards the instances with prediction value larger than 0.5 as positive instances, and the others as negative instances. After updating classifier parameters, the classifier was tested on the withheld test set. Since negative examples far outweigh positive examples in this dataset, XGBoost automatically optimized using the error hyperparameter. Over a set of *E* examples containing *E*_*W*_ wrong examples *E*_*W*_⊂*E*, XGBoost's binary classification error rate ϵ is calculated as

(1) $$\begin{array}{r} \epsilon =E_{W}/E \end{array}$$

We calculated false acceptance and false rejection rates (FAR and FRR, respectively) from these results. Over false attempts *FA* of which some subset *FA*_*S*_ were successful, and true attempts *TA* over which some subset *TA*_*U*_ were unsuccessful:

(2) $$\begin{array}{r} FAR=FA_{S}/FA \end{array}$$

(3) $$\begin{array}{r} FRR=TA_{U}/TA \end{array}$$

To further test the robustness of the system, we also conducted a “leave one out” process for the best performing tasks in which each participant's FAR was calculated once with each other participant left out (e.g., CV for P1 with P2 left out, then CV for P1 with P3 left out, etc., for every participant combination).

## 5. Results

**Figure 4:**
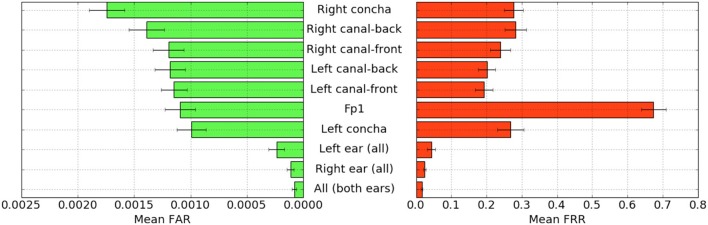
Mean FAR and FRR by electrode configuration across all participants and tasks. All electrodes (Fp1, right, and left ear channels) combined achieved the best FAR score by mean and standard error. The right ear electrodes combined, and left ear electrodes combined, achieved next-best accuracy, both within error of one another.

For each configuration of electrodes, we calculated the mean FAR and FRR across all participants using each task as the passthought (Figure 4). Incorporating all electrodes data resulted in the lowest FAR, followed by the combined right and left ear electrodes, respectively. For left ear (3 electrodes), right ear (3 electrodes), and both ears (6 electrodes) configurations, every participant had at least one task with zero FAR and FRR. Among the individual electrodes, the left canal front electrode produced a mean FAR of 0.12% and a mean FRR just below 20%. Counter to our expectations, Fp1 does not perform as well as most ear electrodes, though overall these reported FAR rates are «1%.

For each position, FAR was about ten times lower than FRR, which is preferable for authentication, as false authentications are generally more costly than false rejections.

Our results indicate acceptable accuracy using data from the left ear alone. This corresponds to a desirable scenario, in which the device could be worn as a single earbud. As such, we focus on results from only the left ear in the following analyses.

### 5.1. Authentication Results

Using only data from the three left ear electrodes, the FARs and FRRs of each task for each participant are shown in Tables 3, 4, respectively. We find at least one task for each participant that achieves 0% FAR, and for five participants a task where both the FAR and FRR are 0%. Each task achieved perfect 0% FAR and FRR for at least one participant, notably *breathe* and *song - open* achieved perfect FAR and FRR for three out of seven participants.

**Table 3:** FAR performance of each task for each participant using data from the left ear.

**Task**	**P1**	**P2**	**P3**	**P4**	**P5**	**P6**	**P7**	**Mean**
Breathe	0	0	0	0	0.0002	0.0004	0	0.00009
Breathe - open	0	0	0	0	0.0002	0	0	0.00003
Face	0	0	0	0.0016	0.0030	0	0.0002	0.00078
Listen	0.0002	0	0.0002	0	0.0026	0	0	0.00043
Sequence	0	0.0002	0	0.0008	0.0014	0	0.0002	0.00037
Song	0	0.0001	0	0	0	0.0001	0	0.00003
Song - open	0	0.0004	0	0	0	0	0	0.00006
Speech	0	0	0.0006	0.0002	0.0002	0.0006	0	0.00022
Sport	0	0	0	0	0	0	0	0

**Table 4:** FRR performance of each task for each participant using data from the left ear.

**Task**	**P1**	**P2**	**P3**	**P4**	**P5**	**P6**	**P7**	**Mean**
Breathe	0	0.0125	0	0.0125	0.0125	0.0250	0	0.00893
Breathe - open	0.0500	0.0125	0.0375	0.1000	0.0375	0	0	0.03393
Face	0.0125	0.0125	0	0.1125	0.4000	0	0.0375	0.08214
Listen	0.0750	0.0375	0.0375	0.0500	0.3375	0.0125	0	0.07857
Sequence	0.0125	0	0	0.0375	0.4000	0.0375	0	0.06964
Song	0.0375	0.0125	0	0.0375	0.0500	0	0	0.01964
Song - open	0.0250	0.0250	0.0500	0.0125	0	0	0	0.01607
Speech	0	0.0125	0.0625	0	0.3375	0	0.0125	0.06071
Sport	0.0250	0.0250	0	0.0125	0.0375	0.0125	0.0125	0.01786

**Figure 5:**
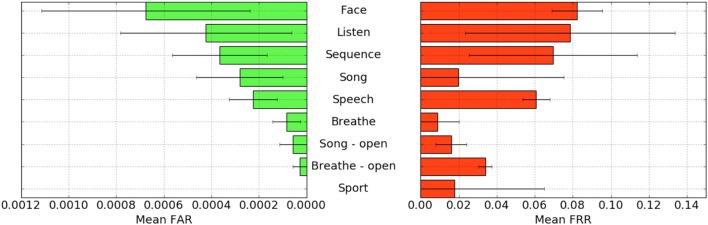
FAR and FRR results by task, across all subjects, using data from the left ear only.

FAR and FRR results by task are shown in Figure 5, averaged across participants. Across all tasks, the sport task produced the lowest FAR. Specifically, it produced 0% FAR for all seven participants, with a corresponding 1.8% FRR. This suggests that the authentication scheme can work very well even if we limit the passthoughts to just a single task category, where the users could choose a personalized secret for that task. Interestingly, tasks like *breathe* and *breathe - open* performed very well despite lacking a personalized secret, indicating that even when the task may be the same across participants our classifier was still able to distinguish between them.

As an omnibus metric, the half total error rate (HTER) is defined as the average of the FAR and FRR:

(4) $$\begin{array}{r} HTER=\left(FAR+FRR\right)/2 \end{array}$$

and from this we estimate authentication accuracy, *ACC*, as:

(5) $$\begin{array}{r} ACC=100*\left(1-HTER\right) \end{array}$$

Using our best performing tasks' FARs, averaging 0% and these tasks' associated FRRs, averaging 0.36%, we obtain an overall authentication accuracy of 99.82% using data from the three electrodes in the left ear. For comparison, if we limit ourselves to only a single electrode (left canal-front), we obtain an authentication accuracy of 90%.

Our “leave one out” analysis with participants' best tasks maintained 0% FAR across all participant combinations.

As an additional validity check, we replicated our results using data from the left ear only, high-passing the original frequency-domain data at 32 Hz to select only data associated with non-cortical signals such as muscular activity. Our classifier performed roughly at chance. This analysis strongly suggests that EMG signals did not significantly contribute to our results. Future work may assess the relative contribution of different EEG frequency bands, as we discuss further in our discussion.

### 5.2. Relative Contributions of Authentication Factors

Our results thus far establish good performance in our default training strategy, in which we count as negative examples recordings from the wrong participant performing any task. We further performed three other analyses with differing negative examples which serve to isolate and test the inherence and knowledge factors: the correct task recorded from the wrong participant (relies on inherence only), the wrong task recorded from the correct participant (relies on knowledge only), and a combination of these two. Positive examples were always the correct participant performing the correct task.

Overall, our default training strategy which engages both knowledge and inherence factors achieves the lowest FAR (Table 5). The FAR in the inherence-only scenario (Table 5, row 2) is ten times higher, and in the knowledge-only scenario (Table 5, row 3) FAR is one hundred times higher, though for all scenarios FAR is less than 1%. However, FRR is *lower* with the inherence-only training strategy than the default. FRR is highest in the combined negative examples case (Table 5, row 4), though FAR remains low.

**Table 5:** Four analyses in which classifiers were trained on differing negative examples paired with resulting mean FAR and FRR across all participants and tasks.

**+ Examples**	**- Examples**	**FAR**	**FRR**
*P*_*c*_, *T*_*c*_	*P*_*i*_, T_*_	0.000074	0.004424
*P*_*c*_, *T*_*c*_	*P*_*i*_, *T*_*c*_	0.000724	0.001522
*P*_*c*_, *T*_*c*_	*P*_*c*_, *T*_*i*_	0.002523	0.039702
*P*_*c*_, *T*_*c*_	*P*_*i*_, T_*_+*P*_*c*_, *T*_*i*_	0.000186	0.052565

P_c_ indicates correct participant, P_i_ incorrect participant, T_c_ correct task, T_i_ incorrect task, and T_*_ any task.

### 5.3. Usability

Before the end of the session, participants completed a usability questionnaire. Participants were asked to rate each mental task on four 7-point Likert-type scales: ease of use, level of engagement, repeatability, and likeliness to use in a real-world authentication setting. Mean ratings across participants for each of these dimensions for each task are shown in Table 6.

**Table 6:** Mental tasks ranked by mean ratings (μ) on 7-point Likert-type scales across participants in four usability dimensions.

**Ease of Use**		**Engagement**	
**Task**	**μ**	**Task**	**μ**
Breathe	6.75	Sequence	5
Listen	6.75	Song	5
Breathe - Open	6.5	Song - Open	5
Song	5.25	Sport	4.75
Song - Open	5	Face	4.5
Speech	5	Speech	4
Sport	3.5	Breathe	2.5
Face	2.75	Breathe - Open	2.25
Sequence	2.25	Listen	2.25
**Repeatability**		**Likeliness to Use**	
**Task**	μ	**Task**	μ
Breathe	7	Song - Open	5
Breathe - Open	6.75	Sequence	4.25
Listen	6.75	Song	4
Song	4.75	Sport	4
Speech	4.75	Breathe - Open	3.75
Song - Open	4.25	Speech	3.75
Face	3	Face	3.5
Sport	3	Listen	3
Sequence	2.5	Breathe	2.75

Participants also ranked the tasks overall from most (1) to least (9) favorite. *Song - open* ranked highest (μ = 4.25) followed by a tie between *breathe - open, song*, and *speech* (μ = 4.75). *Sequence* (μ = 7.75) and *face* (μ = 6.75) were ranked least favorite overall.

In addition to the scales and rankings, we included a few open response questions to ascertain attitudes around use cases for in-ear EEG and passthoughts, and the comfort of wearing an in-ear EEG device in everyday life. Participants first read the prompt, “Imagine a commercially available wireless earbud product is now available based on this technology that you've just experienced. It requires minimal effort for you to put on and wear,” and were asked about use cases for in-ear EEG and passthoughts. Responses about in-ear EEG expectedly included authentication for unlocking a phone or computer and building access, but also aspects of self-improvement such as P4's response “Help people increase focus and productivity.” P5 and P6 also indicated a use for measuring engagement with media like movies and music, and relatedly P4 wrote “music playback optimized for current mental state and feelings.” In terms of comfort wearing such a device, participants generally responded they would be comfortable, though P5 and P6 stipulated only when they already would be wearing something in the ears like earphones. Notably, three participants also added that imagining a face was difficult and had concerns regarding their ability to repeat tasks in the same exact way each time.

A final component of usability we assessed was the ability of the participants to recall their specific chosen passthoughts. Participants were contacted via e-mail approximately 2 weeks after data collection and asked to reply with the passthoughts they chose for the *song, sport, speech, face*, and *sequence* tasks. All participants correctly recalled all chosen passthoughts, with the exception of one participant who did not recall their chosen word component for the *sequence* task.

## 6. Imposter Attack

While our authentication analysis establishes that passthoughts achieve low FAR and FRR when tested against other participants' passthoughts, this does not tell us how robust passthoughts are against a spoofing attack, in which both a participant's custom-fit earpiece, and details of that participant's chosen passthought, are leaked to an imposter who attempts authentication. We performed four different analyses to investigate the system's robustness against imposter attacks.

First, we tested the ability of an imposter to wear an earpiece acquired from someone else and achieve viable impedance values for EEG collection based on the fit of the pieces in their ears. P1 tried on each of the other participants' customized earpieces. The impedances from each electrode were recorded and are listed in Table 7 below. Across all cases, the impedances are not only higher (worse), but also deviate significantly from those achieved by the pieces' intended owners themselves (Table 2). These results come as no surprise given the uniqueness of ear canal shapes between individuals (Akkermans et al., 2005), and point to the possibility that the presentation of a physical token that provides the correct impedance levels can be used as another demonstration of both the inherence and possession factors.

**Table 7:** Electrical impedances with P1 wearing each other participant's (P) custom-fitted earpieces, for concha (C), canal-front (F), and canal-back (B).

	**Impedance [k**Ω**]**					
	**Left ear**			**Right ear**		
**P**	**C**	**F**	**B**	**C**	**F**	**B**
2	34.1	10.2	12.8	27.8	16.0	16.3
3	21.1	20.9	19.0	13.5	11.3	19.5
4	14.1	11.9	9.7	11.0	11.1	13.3
5	17.2	21.9	10.3	32.6	12.5	11.6
6	18.7	10.0	8.4	14.8	11.5	8.9
7	91.5	>1000	21.5	33.5	26.4	31.0

Second, to explore the scenario of an imposter attempting to gain access, we chose the case of the most vulnerable participant, P6, whose earpieces P1, P2, and P7 had the lowest impedances while wearing (Table 8). We collected data using the same data collection protocol, but had the “imposters” refer to P6's list of chosen passsthoughts.

**Table 8:** Left concha (C), canal-front (F), and canal-back (B) electrode impedances of “imposters” P1, P2, P7, and “PX”—a person completely outside of the system—wearing P6's left earpiece.

	**Impedance [k**Ω**]**		
**P**	**C**	**F**	**B**
1	18.7	10.0	8.4
2	46.7	35.7	24.8
7	44.5	20.5	26.3
X	70.0	10.5	8.9

Each imposter performed each of P6's passthoughts (simulating an “inside imposter” from within the system). Following the same analysis steps, we generated 200 samples per task for our imposters, using data from all left ear electrodes.

Since every participant has one classifier per task (for which that task is the passthought), we are able to make 200 spoofed attempts with the correct passthought on each of P6's classifiers. We find zero successful spoof attempts for tasks with a chosen secret (e.g., *song* or *face*). In addition, we also do not find any successful spoof attacks for tasks with no chosen secret (e.g., *breathe*). In fact, in all 1,800 spoof attempts (200 attempts for each of the nine classifiers), we do not find a single successful attack on any of P6's classifiers.

Since this participant's data appeared in the initial pool, the classifier may have been trained on his or her recordings as negative examples. As our third analysis, to explore the efficacy of an outsider spoofing recordings, we repeated the same protocol with an individual “PX” who did not appear in our initial set of participants (an “outside imposter”). Again, we find zero successful authentications out of 1,800 attempts.

Fourth, our “leave one out” analysis can also be seen as another set of outside imposter attacks, in which each participant acts as an outside imposter for each other participant, but where the imposters have their own manufactured earpieces and passthoughts. The best task classifiers achieved FARs of 0% across all combinations, successfully rejecting the simulated imposters.

## 7. Discussion, Limitations and Directions for Future Work

Our findings demonstrate the apparent feasibility of a passthoughts system consisting of a single earpiece with three electrodes, a ground, and a reference, all in or on the left ear. Notably, the gain in performance when adding an additional three electrodes from the right ear is only marginal in our results, suggesting a single earpiece could suffice though this may change with larger sample sizes. FARs and FRRs are consistently low across all participants and tasks, with FARs overall lower than FRRs, a desirable pattern as FAR is the more critical of the two in terms of accessing potentially sensitive information. Participants' best-performing tasks or passthoughts typically see no errors in our testing. From our various training/testing schema it emerged that the inherence factor performs better on its own compared to the knowledge factor, but the combination of the two achieves the lowest FAR indicating measurable benefit of multiple factors. Furthermore, we were able to achieve these results by generating feature vectors based on only 500ms of EEG signal (300 voltage readings across the three electrodes), suggesting that passthoughts can be captured and recognized quickly. Passthoughts also appear to be quite memorable given our 2-week recall follow-up and a few were rated highly repeatable and engaging. Furthermore, no spoofed attacks were successful in our analyses.

Compared against the 80% authentication accuracy achieved with a single generic-fit electrode (Curran et al., 2016), we are able to achieve 90% accuracy with a custom-fit earpiece using data from a single electrode, and 99.8% accuracy with the same custom-fit earpiece using three electrodes. This points to the importance of both the goodness-of-fit of the electrodes and the number of channels as contributors to authentication performance.

These personalized custom-fit earpieces can also be easily outfitted with a hardware keypair for signing authentication attempts, so as to function as a physical token similar to the way an electronic key fob can be used to unlock a car, but with additional inherence and knowledge factors in place.

Several tasks performed exceedingly well among participants, even tasks like *breathe* and *breathe - open* which did not have an explicit secondary knowledge factor as in *song* or *face*. This suggests a passthoughts system could present users with an array of task options to choose from without significant loss in security. While *sport* performed best in terms of low FAR and FRR, it was not rated highly in usability dimensions or as a favorite by our participants. Tasks like *breathe - open* and *song - open* however, both performed well and were rated quite favorably. Interestingly, the *sequence* task was rated low in ease of use and repeatability, and as the least favorite among participants, but was rated highest in likeliness to use in a real-world setting. *Sequence* was arguably the most complex task, and its high rating in likeliness to use could indicate that users are more likely to use a task they perceive as more secure even at the cost of additional effort. This is true afterall for one of the most common forms of authentication, alphanumeric passwords, where increased complexity ensures better performance. The topic of user perceptions of different passthoughts as means of authentication warrants its own research.

The difficulty of stealing someone else's knowledge factor emerged in our spoofing attacks. In conventional password-based systems, once the knowledge factor is divulged, an attacker can essentially spoof the target with 100% success rate. In a passthought-based system, even though our target participant documented their chosen passthought, the spoofers found ambiguity in how these passthoughts could be expressed. For example, for the *face* task, the spoofers did not know the precise face the original participant had chosen. For the *song* tasks, though the song was known, the spoofers did not know what part of the song the original participant had imagined, or how it was imagined. This experience sheds light on passthoughts' highly individual nature and suggests there may be intrinsic difficulty in spoofing attempts. Future work should examine this effect more explicitly to elucidate the effect of knowledge task specificity on defense against imposters.

Performance on Fp1 was not as high as performance in the ear, despite Fp1's popularity in past work on passthoughts (Chuang et al., 2013). One plausible explanation is that several of our mental tasks involved audio (real or imagined), which we would expect to be better observed from the auditory cortex near the ears, as opposed to frontal lobe activity (e.g., concentration) that might be more easily picked up near Fp1. Another possible explanation is that Fp1 may be more sensitive to large, task-irrelevant artifacts from EOG and facial EMG. In either case, future work should continue to investigate what classes of mental tasks best lend themselves to in-ear recording.

The sample size of our study, while small, is comparable to that of other EEG authentication studies (Poulos et al., 2002; Marcel and Millan, 2007; Ashby et al., 2011; Chuang et al., 2013; Curran et al., 2016) and other custom-fit in-ear EEG research (Kidmose et al., 2013; Mikkelsen et al., 2015). The fitting and manufacturing of custom-fit earpieces for each recruited participant was the main limitation to increasing our sample size. This may very well pose a limitation in the proliferation and adoption of such a technology as well, although recently there have been developments in at-home kits for creating one's own custom-fitted earpieces (Voix et al., 2015) that could help overcome this barrier.

The relative homogeneity of our participant pool can be seen as a strength of the reported results, given that system is meant to distinguish between individuals. For future studies however, we should expand the size and diversity of participants, encompassing users and use cases which this system would be particularly applicable such as those with extreme security needs and/or persons with disabilities which may prevent them from performing other authentication methods, e.g., those that require the use of one's hands, voice, or particular bodily movement patterns.

Our work aimed primarily to evaluate our authentication system's security characteristics. As such, we have not investigated which EEG frequency bands drive the authentication results. Future work could re-analyze our data to better understand which frequency bands are most contributing to our authenticator's results. This work would deepen our neuroscientific understanding of how the authentication system achieves the results we observe.

Applications for a system like the one we propose here span any use case for authentication, but some may be particularly well-suited. As has been the motivation for much of the original and ongoing BCI research and development, brain-based systems like this one are nearly universally accessible for use by a wide variety of people with different bodies. As previously mentioned, one's particular passthought is immune to observation and so is apt for use in public spaces or times when malicious observation is likely, and would be extremely difficult to coerce (or even willingly share). To aid in adoption, this system could be aligned with currently used technology of similar form factors, for example speakers could be placed inside our current custom-fit pieces to produce working “hearables” that could be used as ordinary headphones.

### 7.1. Limitations

A key limitation to this work is that our experiments were conducted in a controlled laboratory setting with participants in a stationary, sitting position. Future work should examine EEG data collected from a variety of different user states: ambulatory or distracting settings, during physical exertion or exercise, under the influence of caffeine or alcohol, etc., as well as over longer periods of time or in multiple recording sessions. While these additional conditions may limit the performance of the system, it is interesting to consider which if any limitations might be advantageous in some way. For example, a system that prevents or allows access only when a user is in a certain state of mind or setting, or enforces a biologically-based expiration that requires classifier re-training and thus offers protection in a scenario where a user's original EEG pattern was somehow leaked or surreptitiously stored.

Finally, our work leaves room for some clear user experience improvements. Future work should test the performance of this system using dry electrodes, which are commonly found in consumer EEG devices and have shown recent promise for ear EEG systems (Kappel et al., 2018), as eliminating the need for conductive gel would very likely improve comfort and usability and it is unlikely any system involving gel will be widely adopted. Future work should also attempt a closed-loop (or online) passthought system, in which users receive immediate feedback on the result of their authentication attempt. A closed-loop BCI system would assist in understanding how human learning effects might impact authentication performance, as the human and machine co-adapt.

### 7.2. Health, Neuroscience and In-Ear EEG

Neuroscience fuels some of the most chilling predictions in science fiction (Armstrong and Welsh, 2011). It also stands for some of the greatest possible advances in medicine, mental health, and understanding of human behavior. One ambitious goal is to detect or even predict seizures (Mormann et al., 2006).

However, the original, and most active areas of research in BCI surround the creation of tools for persons with muscular disabilities (Carrino et al., 2012). By collecting unstructured or semi-structured EEG data in the wild, passthought systems could help improve the development of such BCIs (Grierson and Kiefer, 2011). The small size of data repositories, limited mostly by the clinical trials needed to build BCIs for persons with disabilities, has consistently frustrated attempts to improve on algorithms and protocols in this field (Allison, 2009). Although passthought users may not have muscular disabilities, pursuing passthoughts as an area of research will inevitably yield larger repositories of EEG data than have been collected to date. This data could prove invaluable for the development of EEG-based BCIs across a variety of fields, including (but not limited to) assistive technologies.

Again, these opportunities must strike a balance with the risks borne by users around privacy and security. Violating user privacy by revealing EEG data, even to researchers, could undermine any chance of wider BCI adoption in the long-term. Striking this balance will require a deeper understanding of the statistical properties of signals. How much data will users really need to give up? What counts as an “anomalous” reading? Answers to these questions could themselves inform neuroscientific inquiry. This balance will also require a deeper understanding of individuals' attitudes about the meaning of such signals, and how private people believe them to be.

In general, as sensors grow smaller and cheaper, devices more connected, and machine learning more sophisticated, people will build increasingly high-resolution models of human physiology “in the wild.” Passthoughts present just a microcosm of the good such advances might bring, along with some of the most pressing anxieties: What does pervasive physiological recording mean for our privacy, security, safety? The balancing act between these risks and opportunities will prove recurring theme for decades to come. Perhaps passthought authentication could better protect sensitive readings such as EEG. Probing the outer limits of ubiquitous, pervasive sensing can shed light on both the good and bad of ubiquitous physiological monitoring.

## 8. Conclusions and Outlook

Using custom-fit EEG earpieces, we produced a one-step, three-factor authentication system. We demonstrated that our system has high accuracy, higher than prior work using non-custom earpieces. We demonstrated that both inherence and knowledge factors contribute to authentication accuracy, and performed a simulated attack to show our system's robustness against impersonation. We believe that custom-fit EEG earpieces provide a practical path forward for BCI applications, security-related and beyond, both for healthy individuals and for persons with disabilities.

## Ethics Statement

This study was carried out in accordance with the recommendations of the UC Berkeley Committee for Protection of Human Subjects, Biomedical Committee. with written informed consent from all subjects. All subjects gave written informed consent in accordance with the Declaration of Helsinki. The protocol was approved by the Biomedical Committee.

## Author Contributions

NM prepared the final manuscript, contributed to writing the manuscript, and performed data analysis. MC performed experiments with participants, designed experimental protocol, and contributed to writing the manuscript. SG helped to build the in-ear EEG devices, and assisted MC in performing the experimental protocol. JC oversaw the project and contributed to writing and preparing the manuscript.

### Conflict of Interest Statement

The authors declare that the research was conducted in the absence of any commercial or financial relationships that could be construed as a potential conflict of interest.
